# A Case of Hypereosinophilia-Associated Multiple Mass Lesions of Liver Showing Non-Granulomatous Eosinophilic Hepatic Necrosis

**DOI:** 10.4021/gr336e

**Published:** 2011-07-20

**Authors:** Hiroko Ikeda, Kazuyoshi Katayanagi, Hiroshi Kurumaya, Kenichi Harada, Yasunori sato, Motoko Sasaki, Yasuni Nakanuma

**Affiliations:** a Section of Diagnostic Pathology, Kanazawa University Hospital, Japan; b Department of Pathology, Ishikawa Prefectural Central Hospital, Japan; c Department of Human Pathology, Kanazawa University Graduate School of Medicine, Kanazawa, Japan

**Keywords:** Charcot-Leyden’s crystal, Chronic eosinophilic leukemia, Eosinophilia, Liver mass, Visceral larva migrans

## Abstract

Hypereosinophilic syndrome (HES) is defined by elevation more than 1.5×10^9^/L of presence of a peripheral blood count, evidence of organ involvement, and exclusion of secondary eosinophilia such as allergic, vasculitis, drugs, or parasite infection and also clonal eosinophilia. We present the HES case with hepatic involvement. The patient is 70-year-old male. He complained fever and back pain. Blood examination showed marked peripheral eosinophilia, elevation of transaminase and biliary enzymes. Multiple irregular mass lesions of the liver were pointed out by CT and MRI. The liver biopsy was done for differentiation from malignancy. In parenchyma, hepatic necrotic lesion was observed accompanying severe eosinophilic infiltration with Charcot-Leyden’s crystals. There was granulomatous reaction. He was diagnosed as HES and got recovery due to steroid therapy. From the review of HES article, the hepatic histology is categorized into four types as below: 1) cholangitis type; 2) chronic active hepatitis type; 3) vasculopathic type, 4) hepatic necrosis type. Our case is classified in hepatic necrosis type. This type seems to be important to distinguish malignant tumor and also visceral larva migrans by liver biopsy.

## Introduction

Prominent eosinophilic infiltration of the liver is commonly seen in visceral larva migrans histologically presenting palisading granuloma [1, 2]. Occasional eosinophilic infiltration to some degree is known in drug-induced hypersensitivity as well as primary sclerosing cholangitis (PSC) [3], primary biliary cirrhosis (PBC) [4]. Hypereosinophilic syndrome (HES) is indispensable disease related to eosinophilic hepatic necrosis, although it is rare.

Hypereosinophilic syndrome is thought to be a heterogeneous group of disorders characterized by peripheral eosinophilia and involvement of various organs. The diagnosis needs exclusion of other specific causes for eosinophilia such as infectious disease (parasitosis), autoimmune diseases (Churg-Strauss syndrome, Wegener granulomatosis), allergic inflammation to specific antigen, and neoplastic hematopoietic disorders [5]. Hepatic involvement of hypereosinophilic syndrome is uncommon, however, has been suggested to associate with the various type of liver injury. For example, cholangitis [6], chronic active hepatitis (CAH) [7], Budd-Chiari syndrome [8], and nodular regenerative hyperplasia (NRH) [9] has been reported in the patients of hypereosinophilic syndrome.

Herein, we present the case of hypereosinophilia with hepatic eosinophilic necrosis in liver histology and multiple liver mass lesions on radiological findings, which was difficult to differentiate from malignancy.

## Case Report

A 70-year-old Japanese male complained fever and back pain. He was pointed out liver injury on blood examination. Imaging studies revealed multiple space-occupying lesion (SOL) of the liver. He was admitted for the examination of liver SOL. He has no familial history, no medication, no any allergy, animal pet, and habits of eating raw meat. In past history, he suffered cholecystlithiasis several years ago. Laboratory data at admission is shown in Table 1. The counts of white blood cell (WBC) elevated, particularly eosinophil increased markedly up to 62% (13200/µl). IgE RIST was high level and eosinophil cationic protein (ECP) was mildly elevated, which implied allergic or other inflammatory reaction with involvement of the eosiophil in the process [10]. The serologic test for *Toxocara* species was negative. Transaminase and biliary enzyme were moderately increased. Noticeable elevation of tumor markers was nothing, except for soluble interluekin-2 receptor (sIL-2R).

**Table 1:** Laboratory Data at Admission (Normal Range)

Item	Value	Item	Value
RBC	452 × 10^4^ /µl (390 - 520 × 10^4^ /µl)	LDH	654 IU/l (110 - 220 IU/l)
WBC	213 × 10^2^ /µl (33 - 90 × 10^2^ /µl)	CRP	2.7 mg/dl (< 0.3 mg/dl)
Eosinophil	62% (1-5%)	IgE RIST	1457.2 IU/ml (< 400 IU/ml)
Platelet	17.3 × 10^4^ /µl (15 - 35 × 10^4^ /µl)	ECP	33.0 µg/l (< 14.7 µg/l)
AST	110 IU/l (12 - 36 IU/l)	IL-4	(-) (< 15 pg/ml)
ALT	108 IU/l (3 - 32 IU/l)	IL-5	(-) (< 8 pg/ml)
ALP	499 IU/l (114 - 394 IU/l)	IL-6	(-) (< 8 pg/ml)
γ-GTP	64 IU/l (9 - 71 IU/l)	sIL-2R	4483 U/ml (190 - 650 U/ml)
Total bilirubin	0.57 mg/dl (0.1 - 1.0 mg/dl)	DUPAN-2	273 U/ml (< 150 U/ml)
Total protein	6.1 g/dl (6.5 - 8.0 g/dl)	PIVKA-II	14 mAU/ml (< 40 mAU/ml)
Amylase	116 IU/l (58 - 166 IU/l)	CA19-9	(-) (< 37 U/ml)
Creatinine	0.75 mg/dl (0.6 - 1.2 mg/dl)	HBsAg	(-)
BUN	13.7 mg/dl (10 - 20 mg/dl)	HCV-Ab	(-)

ALT, alanine aminotransferase; ALP, alkaline phosphatase; AST, aspartate aminotransferase; BUN, blood urea nitrogen; CA19-9, Carbohydrate antigen 19-9; CRP, C-reactive protein; ECP, Eosinophil cationic protein; γ-GTP, gamma-glutamyl transpeptidase; HBsAg; hepatitis B surface antibody, HCV-Ab; hepatitis C virus antibody, IgE, immunoglobulin E; IL-4, interleukin-4; IL-5, interleukin-5; IL-6, interleukin-6; LDH, lactate dehydrogenase; PIVKA-II, protein induced by vitamin K absence or antagonist-II; Plt, platelet; RBC, red blood cell; sIL-2R, soluble interleukin-2 receptor; WBC, white blood cell.

**Figure 1:**
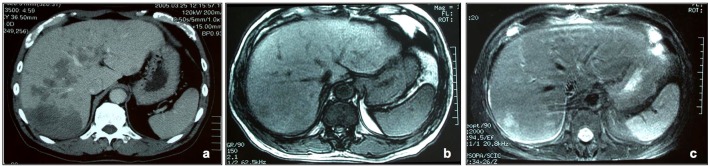
Radiological findings of the liver. (a) Enhanced CT of delayed phase. Multiple low density areas are detected in the whole liver. The lesions show nodular or geographic, and no enhanced effects. (b, c) MRI images. These lesions are iso or low intensity in T1-weight image (b), and iso or hyper intensity in T2-weight image (c).

Radiological finding showed multiple mass lesions of the liver (Fig. 1). Those lesions were low density on abdominal CT, which were not enhanced. MRI image showed low-intensity on T1-weight and iso-intensity on T2-weight. Metastatic tumor, cholangiocarcinoma, malignant lymphoma, and inflammatory pseudotumor were candidates as differential diagnosis. Bilateral pleural effusion was pointed out in extrahepatic organ.

**Figure 2:**
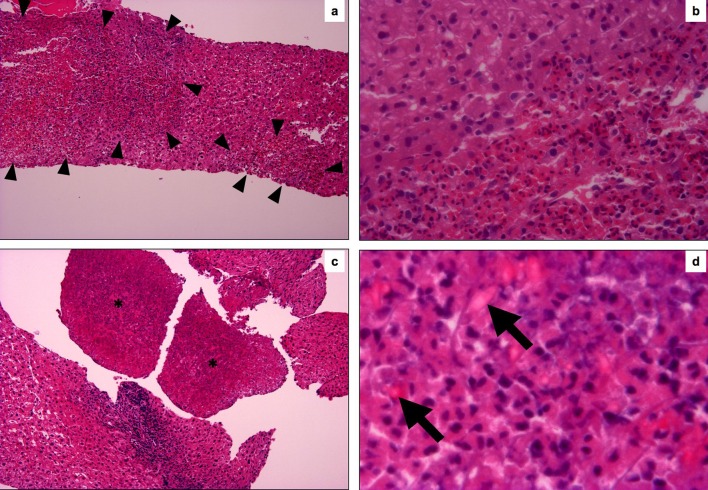
Histological findings of the liver needle biopsy. (a) The foci of eosinophil’s aggregates and hepatocyte’s dropouts are seen in liver parenchyma (surrounded by head of arrow). (HE, original magnification, x100). (b) The border of the eosinophile’s aggregates are relatively clear and cellular atypia of eosinophile are not apparent. (HE, original magnification, x400). (c) Asterisk areas are composed necrotic cells (HE, original magnification, x100). (d) (d) is high magnification of the asterisk area of (c). Charcot-Leyden crystals are scattered in necrotic area, which show lightly eosinophilic and bipyramidal or hexagonal in shape on sections. (HE, original magnification, x1000).

After admission, he complained asthma-like symptom and pleural fluid was detected by chest CT. The liver biopsy was performed for diagnosis of the mass lesions. Liver needle biopsy revealed multifocal eosinophile’s infiltrative lesion in portal tracts and hepatic parenchyma (Fig. 2a, b). Cellular atypia of eosinophile was not distinct. The majority of infiltrative cells were eosinophil in parenchyma, whereas lymphocytes were mixed to some degree in portal tracts. The lymphocytes showed small round nuclei without atypia, and polyclonal on immunohistological findings. The granulomatous reaction was not observed at all. The fragments of necrotic tissue were seen here and there, and a lot of Charcot-Leyden’s crystals were found in the necrotic area (Fig. 2c, d).

The steroid pulse therapy was undergone after liver biopsy. The count of eosinophile in peripheral blood fell down rapidly within normal range after steroid therapy. The hepatic masses became gradually reduced and disappeared in about half a year. He has been followed for 5 years and no recurrence.

## Discussion

Eosinophilia is generally categorized into clonal, secondary, and idiopathic types. Clonal eosinophilia is diagnosed by the presence of histologic, cytogenetic, or molecular evidence of an underlining myeloid malignancy. Actually, a lot of examination including the assessment of peripheral blood smear, bone marrow morphologic features, cytogenetic analysis, mutation analysis of specific gene, lymphocyte phenotyping, and T-cell receptor gene rearrangement are needed to rule out the clonal eosinophilia [11]. Our case is unlikely clonal type because of rapid improvement against steroid therapy, before vigorous examination.

Secondary eosinophilia is caused by parasite infection, allergic, systemic vasculitis, drugs, and nonmyeloid malignancy. In our case, parasite infection, particularly visceral larva migrans, had to be differentiated because hepatic involvement with eosinophilia and multiple lesion on imaging is frequently observed. To our knowledge, the characteristic histological finding of parasitic infection is eosinophilic granuloma in almost cases, except one case of hydatid disease showing eosinophilic cholangitis [1, 2, 12-13]. It seemed that the possibility of visceral larva migrans could be denied because the granulomatous reaction was not found at all in liver biopsy, and antihelminthic treatment was not needed in our patient. Specific factor determining cause of eosinophilia didn’t detected over five years, although it seems to be difficult complete exclusion of secondary eosinophilia.

HES is a subcategory of idiopathic type, and defined by the presence of a peripheral blood eosinophil count of 1.5 × 10^9^/L or greater for at least 6 months, exclusion of both clonal and secondary eosinophilia, evidence of organ involvement, like as Chusid et al. proposed in 1975 [11, 14]. This case didn’t fill strictly above criteria, however, he was good responder of corticosteroid therapy, which is the first-line therapy for HES [5]. A shorter duration by the proper therapy has been allowed for diagnosis for HES [11], therefore, this case likely to be HES.

Liver involvement of the HES does occur in the frequency of 43% [15]. We review the cases reported as HES, which hepatic histology has been evaluated by liver biopsy in Table 2. The variety of histological findings seems reflect the heterogeneity of HES. The hepatic histology of HES is classified into four types: 1) cholangitis type; 2) chronic active hepatitis (CAH) type; 3) vasculopathic type; 4) hepatic necrosis type. Prominent eosinophilic infiltration to portal tracts, bile ducts, venous system, or hepatic parenchyma were observed in each type. The characteristics and differential diagnosis are raised in each group. 1), Cholangitis type (case No. 1 - 5). All patients were man and icteric. The discrimination form PSC were discussed quite often, because similarity of radiological or histological findings, and also complication of eosinophilic colitis, which is consistent with inflammatory bowel disease. 2), CAH type (case No. 6 - 9). Non-specific radiological finding, portal fibrosis with interface eosinophilic hepatitis in histology, and practicable disease control by steroid therapy are characteristics in this groups. PBC, as well as autoimmune hepatitis (AIH) should be excluded by careful clinical follow up and repeated histological examination, because actually AMA-positive, or ANA-positive patient was reported [20, 21]. 3), Vasculopathic type (case No. 10, 11). Case 11 with Budd-Chiari syndrome and FIP1L1-PDGFRA fusion gene is currently categorized into myeloid and lymphoid neoplasm with eosinophilia and abnormalities of platelet derived growth factor receptor α (PDGFRA), PDGFRB or fiibroblast growth factor receptor (FGFR1) in the revised 2008 WHO classification system, and definitely excluded from HES [23]. No abnormalities were detected in chromosomal analysis including Philadelphia chomosome in NRH patients (case No. 10) at the point, but the patients had persisted liver injury over two years despite of steroid, and also hydroxyurea, and thioguanine [9]. Vigorous examination of specific fusion gene and chromosomal abnormalities might be needed whenever encounter the vasculopathic type considering the possibility of neoplastic disease. 4), Hepatic necrosis type (case No. 12, 13). The notable characteristics of this group is hepatic multiple mass lesion pointed out on radiologists, which are difficult to distinguish from malignancy, such as cholangiocellular carcinoma, metastatic carcinoma, or malignant lymphoma [24-26]. Needle biopsy seems essential to exclude above malignancy and also visceral larva migrans, which leads to avoid inappropriate surgery or medication. The patients, just two in reports, both immediately improved against steroid therapy in clinical and radiological findings, which seems to suggest that the group has unlikely neoplastic nature. Drug intake should always remind whenever eosinophilic infiltration is seen in liver, if any pattern.

**Table 2:** Reviewer of the Cases Reported as HES

Case	Age	Sex	Clinical finding	Radiological finding	Pathological finding	Effective therapy	Associated abnormality	Reference
1	28	M	Abdominal cramps, diarrhea, jaundice	Diffuse narrowing and strictures of biliary system (PSC-compatible)	Cholangitis with eosinpophilic infiltration (PSC-like)	Steroid hydroxyurea	Colitis with eosinophilic infiltration	Scheurlen et al [16]
2	41	M	Abdominal pain, fever, jaundice	Stricture and dilatation in extrahepatic bile duct (PSC-compatible)	Eosinophilic sclerosing cholangitis	Steroid		Grauer et al [6]
3	58	M	Jaundice, fatigue, abdominal pain	Normal	Eosinophilic cholangitis	Steroid		Dillon et al [17]
4	20	M	Jaundice, fever	Hepatomegaly, diffuse irregular appearance (PSC-like)	Eosinophilic cholangitis	Steroid, aminosalicylic acid (5-ASA)	Colitis with eosinophilic infiltration (UC-like)	Schoonbroodt et al [18]
5	52	M	Diarrhea, jaundice	NS	Eosinophilic cholangitis	NS	Colitis with eosinophilic infiltration	Sussman et al [19]
6	20	M	Fatigue, myalgia, night sweat	NS	Chronic active hepatitis with eosinophilic infiltration	Steroid	AMA (NS) ANA (+)	Croffy et al [20]
7	34	M	Nausea, jaundice	NS	Chronic active hepatitis with eosinophilic infiltration	Steroid	AMA (NS) ANA (-)	Croffy et al [20]
8	19	M	Jaundice, pruritic rash	Hepatomegaly	Chronic active hepatitis with eosinophilic infiltration	Steroid	AMA (-) ANA (-)	Foong et al [7]
9	65	F	Arthralgias	Normal	Chronic hepatitis with confluent eosinophilic centrilobular necrosis	Steroid	AMA (+), ANA (-)	Ung et al [21]
10	52	M	Malaise, nausea, dizziness, weight loss	Hepatomegaly	NRH, portal eosinophilic infiltration	Steroid, hydroxyurea, thiaguanine	Esophageal varix	Bennie et al [9]
11	27	M	Abdominal fullness	Obstruction of the hepatic veins and stricture of the inferior vena cava (Budd-Chiari syndrome)	Obstructive thrombophlebitis with eiosinophilic infiltration	Interventinal therapy, steroid	Fusion of the FIP1L1 and PDGFRA gene (+)	Inoue et al [8]
12	52	F	Abdominal pain	Liver masses	Hepatic eosinophilic infiltration	Steroid		Lai et al [22]
13	70	M	Back pain, fever	Liver masses	Hepatic eosinophilic infiltration	Steroid		Our case

AMA, anti-mitochondrial antibody; ANA, anti-nuclear antibody; F, female; FIP1L1, FIP1-like 1 gene; M, male; NRH, nodular regenerative hyperplasia; NS, not stated; PDGFRA, platelet derived growth factor receptor α; PSC, primary sclerosing cholangitis; UC, ulcerative colitis.

We presented in this article the case of hepatic eosinophilic necrosis showed liver mass lesions and reviewed the liver histology of HES. In conclusion, the histology is categorized four types: cholangitis type, CAH type, vasculopathic type, and hepatic necrosis type. PSC, PBC, AIH, neoplastic eosinophilia, parasitic infection, malignancy, and drug can be differential disease in each type. It is important that HES is one of the mass forming disease in liver, and liver biopsy is useful to exclude differential disease for steroid therapy.
